# Preparing pharmacists to deliver a targeted service in hypertension management: evaluation of an interprofessional training program

**DOI:** 10.1186/s12909-015-0434-y

**Published:** 2015-09-28

**Authors:** Beata V. Bajorek, Kate S. Lemay, Parker J. Magin, Christopher Roberts, Ines Krass, Carol L. Armour

**Affiliations:** 1 Graduate School of Health, University of Technology Sydney (UTS), Sydney, Australia; 2 Woolcock Institute of Medical Research, University of Sydney (USyd), Sydney, Australia; 3 Discipline of General Practice, University of Newcastle (UNewc), Newcastle, Australia; 4 Sydney Medical School - Northern, Hornsby Ku-ring-Gai Hospital, Sydney, Australia; 5 Faculty of Pharmacy, University of Sydney (USyd), Sydney, Australia

**Keywords:** Hypertension, Pharmacist, Prescribing, Training, Interprofessional, Adherence

## Abstract

**Background:**

Non-adherence to medicines by patients and suboptimal prescribing by clinicians underpin poor blood pressure (BP) control in hypertension. In this study, a training program was designed to enable community pharmacists to deliver a service in hypertension management targeting therapeutic adjustments and medication adherence. A comprehensive evaluation of the training program was undertaken.

**Methods:**

Tailored training comprising a self-directed pre-work manual, practical workshop (using real patients), and practice scenarios, was developed and delivered by an inter-professional team (pharmacists, GPs). Supported by practical and written assessment, the training focused on the principles of BP management, BP measurement skills, and adherence strategies. Pharmacists’ experience of the training (expectations, content, format, relevance) was evaluated quantitatively and qualitatively. Immediate feedback was obtained via a questionnaire comprising Likert scales (1 = “very well” to 7 = “poor”) and open-ended questions. Further in-depth qualitative evaluation was undertaken via semi-structured interviews several months post-training (and post service implementation).

**Results:**

Seventeen pharmacists were recruited, trained and assessed as competent. All were highly satisfied with the training; other than the ‘*amount of information provided’* (median score = 5, “*just right*”), all aspects of training attained the most positive score of ‘1’. Pharmacists most valued the integrated team-based approach, GP involvement, and inclusion of real patients, as well as the pre-reading manual, BP measurement workshop, and case studies (simulation). Post-implementation the interviews highlighted that comprehensive training increased pharmacists’ confidence in providing the service, however, training of other pharmacy staff and patient recruitment strategies were highlighted as a need in future.

**Conclusions:**

Structured, multi-modal training involving simulated and inter-professional learning is effective in preparing selected community pharmacists for the implementation of new services in the context of hypertension management. This training could be further enhanced to prepare pharmacists for the challenges encountered in implementing and evaluating services in practice.

**Electronic supplementary material:**

The online version of this article (doi:10.1186/s12909-015-0434-y) contains supplementary material, which is available to authorized users.

## Background

Hypertension represents a large disease burden in Australia [1] and cardiovascular disease (CVD) is one of the Australian government’s health priority areas [2]. Although hypertension responds well to drug therapy, only 40–60 % of diagnosed hypertensive patients in Australia have their blood pressure (BP) well controlled [3, 4]. This is due to a combination of suboptimal adherence to medications on the part of patients and therapeutic inertia (suboptimal adherence to guidelines) on the part of prescribers [5–7]. In hypertension, the most common non-adherent behavior of patients is discontinuation of therapy [8]. In a review of prescription claim records of nearly 50,000 randomly selected concession card-holders for the 3 years 2004 to 2006, 19 % of patients who had newly prescribed antihypertensive medications did not collect a second prescription [8]. Compared to non-adherent patients, those who are adherent are significantly less likely to develop a cardiovascular event [9].

Community pharmacists are well positioned to address gaps in care of hypertensive patients. Targeted interventions by pharmacists have been shown to improve medicines use [10], the appropriateness of prescribing [11], and BP control in the management of hypertension [12]. Furthermore, pharmacist-led medicines review services significantly contribute to the prescribing of evidence-based therapies in cardiovascular health [13]. Australian pilot studies have also demonstrated the potential for pharmacist prescribing in hypertension management, suggesting that credentialed pharmacists are able to make appropriate therapeutic decisions [14].

However, until such services are fully realized in the Australian health care setting, the pharmacist’s expertise is best utilized currently through collaborative decision-making with clinicians via models of ‘shared care’. The Health Collaboration Model provides an important framework for this, optimizing the respective roles of health professionals whilst providing patient-centered care [15]. A study (funded by the National Heart Foundation of Australia) is underway to explore this within a targeted pharmacist-led service for hypertension management. To fulfill such a role, pharmacists need training in hypertension guidelines and delivery of adherence support strategies. Specially trained community pharmacists can add value to the primary health care management team in terms of medication management. Specifically, therapeutic adjustment recommendations, adherence support and monitoring can all occur in-line with regular visits to the pharmacy.

The purpose of this study was to evaluate a training program designed to enable pharmacists to implement and deliver a targeted service in hypertension management. Specifically, the evaluation canvassed pharmacists’ perspectives on the format of the training program (in terms of structure, duration, quality, content) and how this related to their subsequent preparedness for service provision.

## Methods

**Fig. 1:**
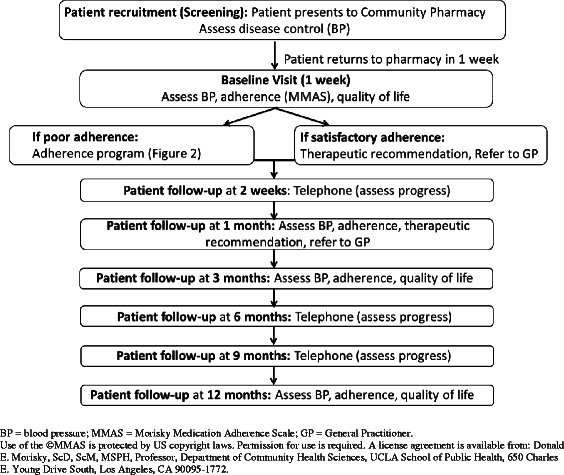
Study protocol for the evaluation of a targeted pharmacist-led service in hypertension management. In this trial, pharmacists recruited to the intervention arm delivered a pharmacist-led service, based on the Health Collaboration Model (HCM) [15]. After initial screening to identify patients with poor BP control, pharmacists optimized a patient’s antihypertensive pharmacotherapy by: systematically identifying the potential cause(s) of poor control; reviewing the use of medicines for hypertension; addressing any adherence issues and/or making therapeutic adjustment recommendations to the patient’s doctor (collaborative care); and monitoring patients at regular intervals (follow-up) to ensure ongoing adherence to medications and assess BP control (Fig. 2)

**Fig. 2:**
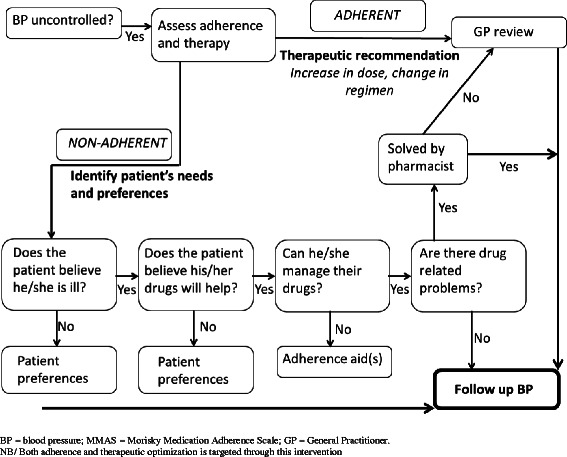
Flowchart of adherence program based on the Health Collaboration Model (HCM)

A training program was developed for community pharmacists who were recruited to participate in an intervention trial evaluating the impact of a targeted pharmacist-led service in hypertension management (Fig. 1; Fig. 2). The training program was evaluated at the time of training as well as after the service had been implemented (i.e., at conclusion of the intervention trial). Conduct of the study was approved by the University of Sydney Human Research Ethics Committee (Application 14483), as well as from the participating Medicare Locals (divisions of general practice).

## The participants

Purposive sampling was used to recruit community pharmacists to be trained for delivery of the intervention (i.e., the pharmacist-led service in hypertension management). Pharmacists were recruited from within each of the two Sydney metropolitan regions where the service was to be piloted in an intervention trial (Northern Sydney and Sutherland Medicare Locals, NSW, Australia), and were selected if they had participated in previous pharmacy intervention studies [16] and whose premises met the following inclusion criteria:

- at least 2 pharmacists on duty to allow the service to be delivered uninterrupted
- a designated quiet and relatively private area for patient education/counselling
- a portable Omron™ BP monitor (with a range of cuff sizes); i.e., an automated BP testing machine which only requires the selection of appropriate cuff sizes and placement for each patient, as well as periodic calibration by the manufacturer

All pharmacists were contacted by telephone (by the project manager) and subsequently provided with an information sheet and consent form, with the aim of recruiting a minimum of 10 pharmacists (maximum 20) to be trained to deliver the service.

## The training program

As a pre-requisite for delivering the service, each pharmacist participated in a tailored training program. Designed according to adult learning principles, i.e., “andragogy”, and social learning theory [17], the training involved components of self-directed learning (i.e., manual of pre-reading), lectures, workshops, case studies, and competency assessment to facilitate self-efficacy in the application of knowledge and clinical skills. The training was provided by the study team which included 3 pharmacy academics and 2 GP academics, with support from 1 project pharmacist (involved in the coordination and management of the trial). A representative from Omron™, whose blood pressure monitors were used in the study, was also present during the practical BP measurement session in order to provide in-depth assistance with use of the devices.

## Training protocol

Pharmacists were provided with clear learning objectives for the training (Table 1) which were based on Bloom’s taxonomy of learning domains (i.e., cognitive, affective and psychomotor domains) [18]. The training comprised several stages as follows: 1) Pre-work manual (based on National Heart Foundation of Australia materials [19, 20]); 2) Training (full-day session comprising a range of targeted activities); and 3) Assessment (Table 2). Regarding the latter, the pharmacists were assessed on their skills in BP measurement (practical assessment), as well as on application of their knowledge to management of patients’ therapy by multiple choice questions and a hypothetical case/scenario (written assessment). They were also given two cases to complete as ‘home work’ and return to (and/or discuss with) the team, to provide additional practice and further build confidence in applying the service protocol. The pass mark was 75 % for all components.

**Table 1:** Summary of learning objectives for the pharmacists participating in hypertension training

Learning objectives were to:	
*Cognitive:* mental skills (knowledge)	1. Appreciate the epidemiology of hypertension
	2. Demonstrate knowledge of evidence-based targets for the management of hypertension including with significant co-morbidities e.g., diabetes.
	3. Be able to demonstrate an advanced understanding of hypertension therapy
	4. Be knowledgeable of appropriate drug treatment and how to monitor it
	5. Understand the barriers to regular medication taking in hypertension
	6. Understand risk assessment and how it is used to choose treatment in hypertension
	7. Have a working knowledge of the health collaboration model to improve medication outcomes
*Affective:* growth in feelings or emotional areas (attitude or self)	8. Justify when to refer patients for follow-up with GP
	9. Reflect on the conclusions drawn from an assessment of the patient’s clinical presentation and therapeutic needs, as a method of on-going professional development
*Psychomotor:* manual or physical skills (skills)	10. Be able to discuss with patients, the roles of age, sex, lifestyle and type of major co-morbidities
	11. Be able to discuss the role of lifestyle changes & anti-hypertensive drugs in lowering blood pressure
	12. Demonstrate how to measure blood pressure accurately and consistently 13. Be able to assess hypertension in practical case study situations and cite realistic interventions
	14. Be able to assess adherence
	15. Demonstrate practical application of adherence strategies with patients on anti-hypertension medications

**Table 2:** Summary of training components for the pharmacists participating in hypertension training

Training manual	Approximately 2 – 3 h of pre-reading (PRE-WORK)		
	Manual of compulsory pre-reading based on National Heart Foundation of Australia materials [19, 20], covering the principle concepts relating to:		
	• Hypertension (background overview): definition, blood pressure and health (complications), epidemiology, regulation of blood pressure (physiology)		
	• Management of hypertension: diagnosis and classification of hypertension, measuring blood pressure, steps to measure blood pressure accurately (including common errors in blood pressure measurement)		
	• Absolute cardiovascular risk: assessing absolute cardiovascular risk in hypertension management, Australian cardiovascular risk charts		
	• Treatment of hypertension: when to intervene in patients with confirmed hypertension		
	• Lifestyle modification: regular physical activity, smoking cessation, dietary modification, weight reduction, limiting alcohol		
	• Drug treatment: general principles, antihypertensive drug classes, selecting an antihypertensive agent, combination therapy, achieving target blood pressure, monitoring response to drug treatment		
	• Treatment considerations in patients with other cardiovascular conditions		
	• Hypertension in pregnancy		
	• Managing inadequate response to treatment		
	• Long-term management		
Training workshop	Full day (including breaks)		
	Topic	Learning mode	Duration
	Introduction	Brief lecture	15 min
	Principles of BP measurement (blood pressure targets, approaches to measurement, salient points in measurement)	Brief lecture, practical workshop	30 min
	Practical BP measurement (practical skills development in BP measurement using real patients and BP device; assessment of skills in BP measurement)	Practice workshop; competency assessment	90 min
	Hypertension management (including optimizing antihypertensive therapy, co-morbidities and considerations for therapy, therapeutic recommendations in practice)	Lecture	45 min
	Medication adherence assessment and intervention (barriers to therapy, how to assess and address adherence, Health Collaboration Model [15]), inter-professional collaboration)	Brief lecture	30 min
	Case scenarios (four hypothetical practice cases which involved identifying individual patient treatment needs, optimal therapy and potential solutions (therapeutic recommendations), assessment of complex patients, supporting patients with multiple co-morbidities)	Workshop, group discussion	30 min
	Case assessment (hypothetical cases scenarios to practice application of above content)	Written assessment	30 min
	Process and documentation for the delivery of the intervention	Workshop	30 min

*BP* Blood pressure

## Evaluation of training

The pharmacists’ experience of the training was evaluated both quantitatively and qualitatively.

### Immediate feedback on day of training

Immediate feedback on the training was sought via a questionnaire (quantitative data), which was administered by the project pharmacist (not the trainers) at the conclusion of the full-day training session. The questionnaire was developed through consensus by the research team, and modelled on others used to evaluate training in similar intervention trials (Table 3). A Likert type scale was used to measure how the training met the pharmacists’ expectations (1 “very well” to 7 “poor”), how different aspects of the training were received (relevance of content to their practice, format of the manual, amount of information provided, workshop activities, duration of the workshop) and how confident pharmacists were to apply the training in practice (1 “extremely confident” to 7 “not at all confident”). The item scores were analyzed (medians, interquartile ranges) using SPSS version 21. The questionnaire also asked several open-ended questions about the factors that enhanced the participants’ learning, and what would help improve their skills, knowledge and ability to apply the skills in the workplace (Table 3).

**Table 3:** Pharmacist evaluation of training

Feedback questions (*n* = 17 pharmacists)	Scale			Median score
To what extent did the training MANUAL meet your expectations?	Very well	1 2 3 4 5 6 7	Very poorly	1 (1 – 2)
To what extent did the training WORKSHOP meet your expectations?	Very well	1 2 3 4 5 6 7	Very poorly	1 (1 – 2)
How would you rate the training MANUAL on the relevance of the content to your practice?	Relevant	1 2 3 4 5 6 7	Irrelevant	1 (1 – 1)
How would you rate the training WORKSHOP on the relevance of the content to your practice?	Relevant	1 2 3 4 5 6 7	Irrelevant	1 (1 – 2)
How would you rate the format^a^ of information provided in the training MANUAL?	Excellent	1 2 3 4 5 6 7	Poor	1 (1 – 3)
How would you rate the amount of information provided in the training MANUAL?	Too little	1 2 3 4 5 6 7	Too much	5 (2 – 5)
How would you rate the amount of information provided in the training WORKSHOP?	Too little	1 2 3 4 5 6 7	Too much	5 (4 – 6)
How would you rate the quality of the training WORKSHOP, specifically the following activities:				
• Lectures	Excellent	1 2 3 4 5 6 7	Poor	1 (1 – 2)
• Technical skills (e.g., blood pressure measurement)	Excellent	1 2 3 4 5 6 7	Poor	1 (1 – 2)
• Case studies	Excellent	1 2 3 4 5 6 7	Poor	1 (1 – 3)
How would you rate the duration of training WORKSHOP?	Too short	1 2 3 4 5 6 7	Too long	5 (5 – 7)
How would you rate your confidence in applying this training into your practice?	Extremely confident	1 2 3 4 5 6 7	Not at all confident	2 (1 – 6)
What factors enhanced your learning?				
What would help you improve your skills?				
What would help you improve your knowledge?				
What would help you improve your ability to apply these skills in the workplace?				
Other comments and suggestions to improve the training?				

^a^“format” refers to structure, organization, and presentation of content

### Feedback collated post-service delivery

One-on-one interviews were conducted several months after the training program, once the pharmacists were delivering the service (intervention) and were able to reflect on the training received. Interviews were conducted by those researchers (pharmacy academics) on the study team who were experienced in qualitative methods. The interviews were facilitated using a semi-structured interview guide (Additional file 1 – Interview Guide), which was principally developed to explore the pharmacists’ experience of delivering the service and their perspectives on chronic disease management, including barriers, challenges, and support needs. The interviews were digitally recorded, then transcribed verbatim by a professional transcription company. The transcripts were thematically analyzed and illustrative quotations that were relevant to the training session were extracted.

## Results

Data were available from seventeen pharmacists who were trained to deliver the service. All pharmacists were assessed as being competent in both the practical assessment of BP measurement and the written assessment.

## Pharmacist feedback on training – questionnaires

All of the pharmacists reported that they were highly satisfied with the education received, with almost all aspects rating the highest score of ‘1’ (Table 3). The only aspect to have not been rated as highly was the ‘amount of information provided’, with the median score being ‘5’ (where 1 = “too little” to 4 = “just right” to 7 = “too much”).

### Format of training

In relation to the educational underpinnings of the training program, the pharmacists indicated that they most valued the team-based approach as well as the inclusion of real patients. The involvement of the GPs was specifically emphasized in the feedback for providing “*helpful insights into doctor’s perspectives*”. The pharmacists felt that the study team provided a “*friendly environment*” and “*good atmosphere*” that was “*interactive*” and conducive to learning, highlighting the approachability between the participants and the study team throughout the whole training. They valued the “*small group*” workshop format, highlighting the ‘hands on’ learning, particularly the “*technical ways of measuring the blood pressure*”. In relation to the delivery of the training program, participants felt that the content was well “*structured*” and “*easy to absorb*”, starting with the background theory (i.e. pre-reading manual, presentation on management principles) and progressing to its practical application in patient care.

Preparation (pre-work) was identified as being integral to the success of the training. The participants commented that the pre-reading manual was very useful to their revision on the management of hypertension, particularly in relation to the use of different pharmacological agents in patients with different co-existing conditions.

### Content

Overall, the feedback from participants suggested that the content addressed through the training was relevant and appropriate, specifically the: management of hypertension (pharmacotherapy, particularly the use of combination medications); correct use of the BP monitoring device; proper technique and process for measurement of BP; and real life application and verification of skills. The two aspects that were most appreciated by respondents were the:

- *BP measurement workshop* – Whilst the participants appreciated the hands-on practice of BP testing using the digital devices, what they particularly welcomed was the involvement of real patients as well as the feedback from GPs as part of the verification of technique (this particularly helped instill confidence in the pharmacists’ skills). The ability to practise their skills during the training session was viewed as highly important with several participants commenting on the need for continual practice post training.
- *Case studies* – these were viewed as highly important to application of knowledge to patient management. In workshopping these cases the participants also valued the interaction and contribution of the team particularly the GPs. Whilst there was effective discussion in relation to the cases, a couple of participants commented that the inclusion of role plays may have further enhanced their learning here.

At this stage of the evaluation, none of the participants specifically commented on the *adherence content* presented throughout the training, with the exception of one participant who thought that the “*more detailed approach on confronting adherence*” enhanced their learning. Overall, it is unclear from the participant feedback how most participants perceived this particular aspect of the training, given the emphasis placed on the BP measurement and management in their feedback.

### Suggestions for improvement of training

One practical approach suggested was the opportunity for “*on-site trainers*” to be available “*to help motivation*” (i.e., motivate the pharmacists to recruit patients) particularly for the “*first few patients*”. A potential “*workshop withlocalGPs*” was also flagged by one pharmacist.

When asked what would help improve their skills and apply them in the workplace, pharmacists noted that more practice, and possibly a refresher course in addition to the workshop they had attended, would be useful. One pharmacist wrote that the practical act of taking the time and measuring patients’ blood pressure would reinforce the skills they had gained in the training, in addition to having feedback on the process: “*actually taking BP measurements and feedback from GPs*”. A couple of pharmacists felt that using the National Heart Foundation of Australia website as a resource, revising the disease state and medications and “*keeping up-to-date with new guidelines, new antihypertensive meds and attending face-to-face lectures on CVD, hypertension etc*” would help to reinforce their knowledge in the area. Overall, only one participant expressed a preference for running future training spread over two days during the week.

### Pharmacist feedback on training – qualitative interviews

One-on-one interviews were conducted several months after the training program, once the pharmacists were delivering the service (intervention) and were able to reflect on the training received. Three key themes in regards to the training emerged: professional development of pharmacy staff, training to deal with unexpected challenges, and impact on pharmacist confidence.

### Professional development of pharmacy staff

Several participants recognized the importance of broader staff training and education to enable them to support and promote chronic disease management services offered in the pharmacy.

*“not just the pharmacist but the pharmacy assistants need to be competent in doing these monitoring services and be able to explain the basic meaning of what these results could indicate to consumers.”* (Pharm 1)

### Training to deal with unexpected challenges

A few pharmacists mentioned some of the unexpected challenges they faced during the delivery of the service, mostly during the patient recruitment stage, unrelated to their therapeutic knowledge and skills. Although for most pharmacists, patient recruitment strategies mainly relied upon using systems already in place in the pharmacy, a few specifically commented on the difficulty of sustaining motivation during recruitment due to competing activities and unexpected incidents, suggesting a need for additional training to address these specific challenges.

*“In the beginning it was very, very easy. We were very excited and very motivated initially to recruit… The problem is that as we progressed, the motivation decreased and of course there are other more urgent pharmacy initiatives that have taken our attention.” (Pharm 2)*

*“… was quite motivated and thought how easy will this be. We did need [patients] to have a blood pressure test to qualify. I got a few unusual (low) readings from people and that put negativity in my mind (about the ability to recruit enough patients).” (Pharm 3)*

None of the pharmacists specifically mentioned any difficulties in recruiting patients due to barriers presented by the GP.

### Impact of training on pharmacist confidence

All except one respondent reported that the study’s training had raised their level of confidence in providing the hypertension management service, with several suggesting becoming more confident in providing chronic disease management services more broadly. Most attributed this confidence to the comprehensive training for participation in the study, as well as the excellent resource packs provided as a source of reference throughout the program.

*“I think the training provided some good materials and we were very confident after the training. The training itself was very well done.” (Pharm 2)*

Two pharmacists commented on improved confidence in specific aspects of their practice, separate to confidence in delivering the service. One respondent said it boosted her confidence in dealing with hypertension in a different way, since it refreshed past knowledge*:*

*“I was really starting to feel like I’ve drifted out of the whole hypertension, feeling confident about it… [It] was like “right, it’s time for me to get back on top of all of this.” (Pharm 4)*

One participant was an intern pharmacist, who felt that the training had particularly boosted their confidence as an early career pharmacist, particularly in relation to collaborating with other health professionals:

*“For me it was quite difficult. I'm only in my first year out as a pharmacist. So telling the doctor what to do was quite difficult. I found that quite hard. It was a good learning experience. I gained a lot from being able to talk to other health professionals and, yes, having the confidence to be able to do that. The further through the study we went, the more and more confident I became in maybe offering the advice to the doctor, not so much telling them what to do but just, yes, giving them some advice on what we believed was the best approach on helping the patient with their hypertension.” (Pharm 5)*

Overall, most pharmacists indicated that the training had boosted their confidence in providing this type of service, however, one participant remained ambivalent about the level of responsibility such a role would give her within the parameters of this shared model of care:

*“[I was] reasonably confident [in making therapeutic recommendations] and we certainly had those notes from the training as well. I’ve been to lots of other trainings on cardiovascular. It is only a recommendation so it’s whether they follow the advice or not …. yes, I would feel confident… whilst you make a recommendation, the onus is on the doctor…” (Pharm 3)*

Only one respondent specifically expressed that they did not feel entirely confident “*self-managing*”, i.e., making independent recommendations, because *“some of them [patients] were on all the recommended medications and I still didn’t know why their blood pressure was high”* and despite following the service protocol, including an adherence assessment (Pharm 6). This respondent commented that anything beyond recommending a review of the patient’s health was beyond the scope of their university level training.

## Discussion

Overall, the positive findings highlighted the utility of this model of training for pharmacists who are seeking to advance their practice. Specifically, the integrated, multidisciplinary, and educational approaches used here were able to engage pharmacists, address their learning objectives, and provide them with the appropriate knowledge and skills to deliver targeted services for hypertension management.

The delivery of an educational intervention that was not resource-intensive (relative to the ≥ 2 day training provided in many intervention trials, as experienced across the study team), and which aligned with the time constraints of these pharmacists (busy practitioners and service providers), contributed to its success. The favorable ratio of study participants to trainers was enabled by the engagement of all research team members (who helped develop the training); however, in future the training could be delivered with fewer trainers if resources were limited. Overall, the training comprised some preparatory reading and participation in a 1-day training program. The time burden was not onerous, with required components being offered in a flexible learning mode (i.e., preparatory readings at home) and/or outside of standard business hours (or, for those pharmacies open on weekends, when it is easiest to find locum pharmacists to cover work-shifts). The importance of these aspects cannot be underestimated; previous studies have shown that a key barrier to pharmacists’ participation in continuing professional development activities is time constraints, with pharmacists preferring modes of education that are consolidated (structured and focused in terms of content), delivered in manageable allotments of time, and which offer some ability for flexible, self-directed learning [21, 22].

Following from this, this model of training emphasized the use of resources (i.e., online practice guidelines, the pharmacist’s own BP monitors) that were already familiar to the pharmacists, enabling them to explore their functionality and clarify their optimal use through application to case scenarios and patients (as part of the training). Overall, this helped pharmacists to better utilize existing and accessible information and tools. Importantly, this approach helped to simulate the practice/service delivery that the pharmacists were being trained for, resulting in a high level of engagement from the pharmacists, clarity about training outcomes, and greater confidence instilled following the training. Indeed, learning modes which encompass elements of simulation have been shown to improve outcomes in terms of communication and teamwork [23], as well as clinical decision-making [24]. In the context of BP management, simulated learning has improved the accuracy of BP measurement [25]. Simulation of real-world practice is therefore important, and our model of training has achieved that through the use of real-life ‘patients’, ‘hands-on’ practice with devices, use of existing practice-based resources, hypothetical cases to reinforce the application of content knowledge, as well as engagement with GPs to discuss real-world practice.

One of the most highly valued aspects of this educational intervention was the ability of pharmacists to directly engage with GPs throughout all stages of workshop training (all workshops, practicals, discussions). Not only did this convey the GPs’ support for the pharmacist’s advanced role, further elevating confidence, it also allowed pharmacists to explore patient care issues and sensitivities in a practical way. Inter-professional practice (IPP) is widely recognised as being integral to patient-centred care, and is being reinforced through inter-professional education (IPE) [26, 27]. Whilst the focus of IPE has, to date, been on the training of university students (undergraduate training) [28], limited research has been done on IPE in practising health professionals. Innovative models of IPE have been developed to engage students of pharmacy and medicine in the quality use of medicines [29], but this has not extended to the professional development of pharmacists and doctors in current practice. Our study identifies the potential for adopting IPE in the professional development of pharmacists, not only to reflect multidisciplinary practice, but in readiness for advanced practice. Furthermore, IPE is important for other health disciplines in clarifying their understanding of the pharmacist’s role, including their specific expertise and training. Studies have reported that, in the context of pharmacist prescribing, GPs remain unaware of the training and activities of community pharmacists, and that any barriers between GPs and community pharmacists could be overcome through greater inter-professional liaison between the two professions [30].

For future training programs, some areas that may need further attention were identified in this study. In particular, there is a need to integrate more closely the pharmacist’s pre-existing knowledge with the new skills being developed. For example, in the present study, there was an assumption that pharmacists would be relatively confident in addressing patient adherence to medication, given that this is often encountered in usual care. However, post-implementation of the service, the pharmacists expressed a need for additional learning in this area to help demonstrate optimal practice and to explore various scenarios and/or strategies, as well as to help integrate all of the intervention (service) components. This can easily be addressed through ‘live’ patient role-plays [31], focusing on behavior change counseling and motivational interviewing [32]. Furthermore, pharmacists identified that service provision in their pharmacies involved other staff (not just the pharmacist), and that future training would benefit from including pharmacist interns and pharmacy assistants.

In addition to aspects of patient care, pharmacists felt a need for additional training in how to efficiently manage a pharmacy-based study, particularly in regard to patient recruitment. In Australia, there is limited formal research training offered to most pharmacists. Internationally, some degree and clinical residency programs incorporate structured research training (e.g., USA), whilst other professional organizations are promoting initiatives to make pharmacists “research ready” [33]. There is clearly a need to up-skill pharmacists in this area.

In considering the findings of this study, it is important to acknowledge the limitations. First, the inclusion criteria may have selected out highly motivated pharmacists with a higher baseline level of knowledge and skill in service delivery, and may therefore not be entirely representative of all community pharmacists. In this regard, the training needs of the wider pharmacist population may vary to those reported here. However, the pharmacies themselves were typical of those encountered in the Australian community setting. Second, evaluation of the training (the qualitative evaluation post-service delivery) was undertaken by members of the study team who had also delivered components of the training, and therefore it is possible that social desirability bias influenced the pharmacists’ responses. Third, whilst the training was not considered to be resource-intensive in this context, it may be more difficult to scale-up the training to larger, less experienced groups of pharmacists, with reduced access to experienced trainers (including inter-disciplinary experts). Nevertheless, the findings provide some useful insights into the training requirements for the delivery of pharmacist-led services.

## Conclusion

Structured, multi-modal training involving simulated and inter-professional learning is effective in preparing selected community pharmacists for advanced practice and the implementation of new services in the context of hypertension management. This training was sufficient to give pharmacists competency in BP measurement and providing therapeutic recommendations to GPs for medication management of hypertension, but could be further enhanced in addressing adherence issues in patients through simulated learning as well as specific training to prepare pharmacists for the challenges encountered in implementing and evaluating services in practice.

## Additional file

Additional file 1: **Interview Guide.** (PDF 16 kb)
