# Supplementary material for: Preparing pharmacists to deliver a targeted service in hypertension management: evaluation of an interprofessional training program
Source: BMC Med Educ. 2015 Sep 28;15:157. doi: 10.1186/s12909-015-0434-y (PMC4587878; doi:10.1186/s12909-015-0434-y)
Supplement: Additional file 1: — Interview Guide. (PDF 16 kb) [file 12909_2015_434_MOESM1_ESM.pdf]

<u>Interview guide</u>

- How do you think community pharmacists can best support people with chronic diseases, especially hypertension?
    - *Examples?*
- In your opinion what role should providing chronic disease management play in your future professional practice?
- What do you think the impact of providing chronic disease management on your business will be?
    - *Positives?*
    - *Negatives?*
- What kind of support would you need to engage in chronic disease management?

On coming to our study …

Intervention and Control groups

- What *was your experience in recruiting patients*
    - *easy or difficult*
- What strategies did you use?
- Which were
    - *successful?*
    - *unsuccessful?*
- Can you suggest things that might have helped?
- Why do you think patients agreed to participate?
- What do you think are some barriers to engaging patients with the study?
- Some patients were initially found to have uncontrolled BP and then came back with controlled BP. What do you think might have caused this change? Did you do anything different with these patients?

Intervention group only

- In the service you were required to provide adherence support and/or therapeutic recommendations for the GP. How confident were you in making these recommendations after training?
- How well do you think your recommendations were received by the GP?
- What kind of barriers did you find/what enabled the communication?
- There has been a lot of focus on pharmacist prescribing in Australia recently. What do you think is needed to facilitate this being implemented in community pharmacies?

Intervention and Control groups

- What is the nature of the relationship between the community pharmacist and GP generally?
- How do you think this relationship might be improved or enhanced?
- What impact, if any, did your participation in this study have on your professional relationship with your local GPs?
